# Dietary taurine intake, nutrients intake, dietary habits and life stress by depression in Korean female college students: a case-control study

**DOI:** 10.1186/1423-0127-17-S1-S40

**Published:** 2010-08-24

**Authors:** Ji-Yeon Park, Jeong-Soon You, Kyung-Ja Chang

**Affiliations:** 1 Dept. of Food and Nutrition, Inha University, Incheon 402-753, Korea

## Abstract

**Background:**

The purpose of this study was to investigate the dietary taurine intake, nutrients intake, dietary habits and life stress by depression in Korean female college students.

**Methods:**

In this study, research data were collected in March 2009 and 65 patients with depression and 65 controls without depression participated. The CES-D (Center for Epidemiologic Studies Depression) scale was used for depression measure and controls were matched for age. A 3-day recall method was used for dietary assessment (2 weekdays and 1 weekend day).

**Results:**

Average height, weight and body mass index (BMI) were 161.3±0.5cm, 55.3±1.0kg and 21.2±0.4kg/m^2^ for depression patients and those of control group were 161.4±0.7cm, 53.1±0.8kg and 20.3±0.2kg/m^2^, respectively. Average dietary taurine intakes of depression patients and control group were 89.1 and 88.0 mg/day, respectively. There was no significant difference in dietary taurine intake between depression patients and control group. The average intakes of vitamin A (p<0.05), β-carotene (p<0.01), vitamin C (p<0.05), folic acid (p<0.05) and fiber (p<0.05) of depression patients were significantly lower compared to control group. The average total dietary habit score of depression patients (47.2) was significantly lower than that of control group (51.3) (p<0.01). The average dietary habit scores of “eating meals at regular times” (p<0.05), “eating adequate amount of meals” (p<0.05), “having meals with diverse foods” (p<0.05), “avoiding eating spicy foods” (p<0.01) and “eating protein foods such as meat, fish, eggs, beans more than 2 times a day” (p<0.05) were significantly lower in depression patients compare to control group. The average scores of total life stress (p<0.001) and all stress categories of depression patients were significantly higher than those of control group except faculty problem score.

**Conclusions:**

These results show that depression patients have poor dietary habits and unbalanced nutrition status. Also depression patients have higher life stress score.

Therefore, continuous nutrition education and counselling for good dietary habits and balanced nutrition status are needed to prevent depression in Korean college students.

## Background

Depression is an usual emotional disability that leads to distress and seriously damages daily functioning [1,2]. Depression is typically more common in women than men [3] because women have higher stress levels and lower achievement levels than men in gender roles [4]. Depression in the college student is a significant problem [5], and depression students have problems in academic work and motivation [6].

Overall nutritional status and nutrients intake are associated with depression, and poor diet quality is the leading causes of depression [7]. A study of childhood showed that food insufficiency has been connected with behavior problems such as depression, aggression and anxiety [8]. It was reported that higher depression level is associated with less regular meals in the adolescents [9]. Depression is closely associated with stress. The stress situation can cause or aggravate depression [10], and adolescent girls respond to general stressors with greater depression [11]. Taurine is an important role as an inhibitory neurotransmitter [12] and is essential for neuronal growth in human body [13]. Taurine is associated with emotional disturbances such as depression [14] and anxiety [15]. It was reported that plasma level of taurine was increased in depression patients [16] and in schizophrenia [17]. However, little information is available on the relationship between depression and dietary taurine intake in humans. Therefore, the purpose of this study was to investigate the relation among depression, dietary taurine intake, nutrients intake, dietary habits and life stress in Korean female college students.

## Methods

### Subjects

Research data were collected in March 2009 and the subjects were 130 female college students residing in the Incheon area. A case-control study was conducted using a questionnaire. The depression group was composed of 65 female college students with depression, and the control group consisted of 65 female college students without depression. Depression was measured by the Korean version of CES-D (Center for Epidemiologic Studies Depression) scale [18]. The CES-D scale, a 20-item self-reporting measure, is 4-point scale ranging from 0 to 3 with a total score of 0-60. Depression is defined as CES-D score of 16 and healthy controls were matched for age. Greater and higher scores indicated higher depressed levels. Subjects submitted to the researcher a written and signed informed consent form to take part in this survey.

### Questionnaire

The dietary habits questionnaire included 16 items which were based on previous studies [19,20]. Each item responses ranged from 1 to 5 and the scores of items negatively worded were reversed before summation. The life stress scale contained 50 items about their experience frequency and  importance of stress, and the response to each item was scored on a 4-point scale from 0 to 3 during the past year [21]. The total life stress score was calculated by multiplying frequency of stress experienced and importance of stress. The higher stress scores are indicative of greater frequency of stress experienced and importance of stress.

### Anthropometric measurement and body composition

The subject’s height was measured using a stadiometer. Measurement of weight, calculation of body mass index (BMI), soft lean mass, body fat percentage, waist-hip ratio (WHR) and calculation of basal metabolic rate (BMR) were measured with the InBody 3.0 Body Composition Analyzer (InBody 3.0, Biospace, Seoul, Korea).

### Intakes of dietary taurine and nutrients

A 3-day recall method was used for dietary assessment (2 weekdays and 1 weekend day). Dietary taurine and nutrients intakes were estimated using the computer-aided nutrition program (CAN-pro 3.0, The Korean Nutrition Society, Korea) inputed with a taurine content database for 17 food groups, commonly used 321 food items [22,23].

### Statistical analysis

The statistical analysis was conducted using the SPSS 12.0 program. Mean and standard error were calculated for all variables. The data obtained between depression group and control group were compared by Student’s t-test.

## Results

### General characteristics

General characteristic data of the subjects are shown in Table 1. The average height, weight and BMI were 161.3cm, 55.3kg and 21.2kg/m^2^ for depression patients and those of controls were 161.4cm, 53.1kg and 20.3kg/m^2^, respectively. According to the 4th Korea National Health and Nutrition Examination Survey, the average height, weight and BMI of 19-29 years old women were 161.4cm, 56.4kg and 21.6kg/m^2^, respectively [24]. The average height, weight and BMI of our subjects were similar to those of the same age group in Korean women. There was significant difference in BMI between depression patients and control group. In the previous studies [25,26], depression was significantly associated with higher BMI in women. Also it was reported that a higher BMI may lead to depression in males but the relationship may not be the same in females [27].

**Table 1:** General characteristics of the subjects

Variables	Depression patients (n=65)	Control group (n=65)
Age (years)	20.6±0.2	20.5±0.2
Height (cm)	161.3±0.5	161.4±0.7
Weight (kg)	55.3±1.0	53.1±0.8
BMI (kg/m^2^)	21.2±0.4^*^	20.3±0.2
Soft lean mass (kg)	36.9±0.5	36.3±0.5
Body fat percentage (%)	28.7±0.6	27.2±0.5
WHR	0.81±0.0	0.80±0.0
BMR	1398.6±12.7	1383.0±13.6

Values are mean ± SE; BMI: body mass index; *: p<0.05, **: p<0.01, ***: p<0.001 (by Student’s t-test); WHR: waist-hip ratio; BMR: basal metabolic rate

### Intakes of dietary taurine and nutrients

Average dietary taurine intake of depression patients and control group was 89.1 and 88.0 mg/day, respectively (Table 2). The daily taurine intake of female college students in Korea was 96.9 mg/day [28]. There was no significant difference in dietary taurine intake between depression patients and control group. It was reported that serum concentrations of taurine was also not significantly different between depression patients and control group [29]. Nutrients intakes of the subjects are shown in Table 3. The average intakes of vitamin A (p<0.05), β-carotene (p<0.01), vitamin C (p<0.05), folic acid (p<0.05) and fiber (p<0.05) of depression patients were significantly lower compared to control group. The average intakes of carbohydrate, vitamin B_6_, vitamin E, iron, zinc, sodium and cholesterol of depression patients were lower compared to control group, but not significantly. It was reported that iron, zinc, and selenium intakes were deficient in depression patients [7]. In addition, folic acid and vitamin B_12_ deficiency were associated with depressive disorders and the patients with severe depression had  low folic acid and low vitamin B_12_ levels in blood [30].

**Table 2:** Dietary taurine intake of the subjects

Variables	Depression patients (n=65)	Control group (n=65)
Taurine (mg/day)	89.0±8.5^NS^	88.0±7.5

Values are mean ± SE; NS: not significant

**Table 3:** Nutrients intake of the subjects

Variables	Depression patients (n=65)	Control group (n=65)
Energy (kcal/day)	1524.5±46.5	1488.4±42.2
Carbohydrate (g/day)	200.2±6.7	203.7±6.2
Total fat (g/day)	52.6±2.0	50.7±2.0
Total protein (g/day)	59.2±2.2	58.6±1.9
Vitamin A (㎍ RE/day)	539.5±27.6^*^	641.3±27.0
β-carotene (mg/day)	2076.6±151.5^**^	2656.0±131.1
Vitamin B_1_ (mg/day)	1.0±0.0	1.0±0.0
Vitamin B_2_ (mg/day)	0.9±0.0	0.9±0.0
Vitamin B_6_ (mg/day)	1.4±0.1	1.5±0.1
Niacin (mg NE/day)	13.4±0.6	12.9±0.4
Vitamin C (mg/day)	52.4±3.2^*^	66.2±4.9
Vitamin E (mg/day)	11.2±0.5	11.9±0.5
Phosphorous (mg/day)	793.8±26.1	776.1±22.5
Calcium (mg/day)	409.6±17.5	372.1±16.2
Iron (mg/day)	9.6±0.4	9.9±0.4
Zinc (mg/day)	6.7±0.2	6.9±0.2
Folic acid (㎍/day)	151.8±6.5^*^	172.1±7.7
Fiber (g/day)	12.1±0.4^*^	13.5±0.5
Ash (g/day)	12.5±0.4	13.4±0.5
Potassium (mg/day)	1794.4±62.1	1788.8±63.2
Sodium (mg/day)	2874.2±115.4	3017.4±123.0
Cholesterol (mg/day)	268.8±14.2	295.7±15.1

Values are mean ± SE; *: p<0.05, **: p<0.01, ***: p<0.001 (by Student’s t-test)

### Dietary habits

Table 4 shows the average dietary habit scores of depression patients and control group. There was significant difference in the average total dietary habits score between depression patients and control group; those of depression patients and control group were 47.2 and 51.3, respectively (p<0.01). The average scores of “eating meals at constant times” (p<0.05), “eating adequate amount of meals” (p<0.05), “having meals with diverse foods” (p<0.05), “avoiding eating spicy foods” (p<0.01) and “eating protein foods such as meat, fish, eggs, beans more than 2 times a day” (p<0.05) were significantly lower in depression patients compared to control group. It was reported that female high school students with depression had poor food behaviour [31].

**Table 4:** Dietary habits scores of the subjects

Variables	Depression patients (n=65)	Control group (n=65)
Eating breakfast regularly	3.3±0.2	3.4±0.2
Eating meals at constant times	2.7±0.1^*^	3.1±0.1
Eating meals slowly	2.8±0.2	3.1±0.1
Eating adequate amount of meals	3.4±0.1^*^	3.7±0.1
Eating three meals a day	2.8±0.2	3.1±0.2
Having meals with diverse foods	3.1±0.1^*^	3.4±0.1
Avoiding overeating everyday	2.6±0.1	2.9±0.1
Avoiding eating salty foods	2.8±0.2	3.2±0.1
Avoiding eating spicy foods	2.5±0.2^**^	3.1±0.2
Eating foods such as meat, fish, eggs, beans more than 2 times a day	2.9±0.1^*^	3.3±0.1
Avoiding eating foods containing oil or mayonnaise more than 2 times a day	3.7±0.1	3.7±0.1
Eating dairy foods(milk, yogurt, etc.) everyday	3.2±0.2	3.0±0.2
Eating greenish yellow vegetable every meals	2.9±0.1	3.1±0.1
Eating fruit everyday	2.8±0.1	3.0±0.1
Avoiding eating sweet foods(snack, chocolate, etc.) everyday	3.1±0.1	3.1±0.1
Applying nutrition knowledge to daily life	2.9±0.0	3.2±0.1
Total score	47.2±0.9^**^	51.3±0.9

Values are mean ± SE; *: p<0.05, **: p<0.01, ***: p<0.001 (by Student’s t-test)

### Life stress

Life stress levels of the subjects are shown in Table 5. The average scores of total life stress (p<0.001) and all stress categories of depression patients were significantly higher than those of control group except faculty problem score. It was reported that depression in middle-aged Korean women was significantly associated with psychological stress and physical stress [32].

**Table 5:** Life stress scores of the subjects

Variables	Depression patients (n=65)	Control group (n=65)
Interpersonal relationship stress		
Faculty problem	7.5±1.3	5.6±1.1
Lover problem	7.5±1.6^*^	3.3±0.9
Friend problem	5.7±1.1^**^	1.9±0.6
Family problem	12.5±2.3^**^	3.9±1.1
Task-related stress		
Grade problem	29.5±2.8^*^	19.7±2.5
Future problem	32.8±2.9^***^	16.4±1.8
Economy problem	24.4±3.2^***^	7.9±1.3
Value problem	26.6±2.7^***^	10.5±1.8
Total score	87.0±6.3^***^	41.7±4.2

Values are mean ± SE; *: p<0.05, **: p<0.01, ***: p<0.001 (by Student’s t-test)

## Conclusions

Our study investigated the relation among depression, dietary taurine intake, nutrients intake, dietary habits and life stress. The average intakes of vitamin A, β-carotene, vitamin C, folic acid, fiber of depression patients were significantly lower compared to control group. The average total dietary habit score of depression patients was significantly lower and total life stress score of depression patients was significantly higher compared to control group. However, there was no significant difference in dietary taurine intake between depression patients and control group. These results show that depression patients have poor dietary habits and unbalanced nutrition status. Therefore, continuous nutrition education and counselling for good dietary habits and balanced nutrition status are needed to prevent depression in Korean college students.

## Abbreviations

 CES-D: Center for Epidemiologic Studies Depression; BMI: body mass index; WHR: waist-hip ratio; BMR: basal metabolic rate

## Competing interests

The authors declare that they have no competing interests.

## Authors' contributions

JYP carried out the data collection, performed the statistical analysis and drafted the manuscript. JSY participated in its design and coordination and helped to draft the manuscript. KJC supervised the design and execution of the study. All authors read and approved the final manuscript.
